# Intraoperative Flow Cytometry for the Differentiation of Glioblastomas from Metastatic Brain Tumours: A Preliminary Prospective Study

**DOI:** 10.3390/cancers18162661

**Published:** 2026-08-18

**Authors:** George A. Alexiou, Marios Lampros, Georgios Markopoulos, Spyridon Voulgaris, George Vartholomatos

**Affiliations:** 1 Department of Neurosurgery, School of Medicine, University of Ioannina, 45500 Ioannina, Greece; 2 Haematology Laboratory-Unit of Molecular Biology and Translational Flow Cytometry, University Hospital of Ioannina, 45500 Ioannina, Greece; 3 Department of Physiology, Faculty of Medicine, School of Health Sciences, University of Ioannina, 45110 Ioannina, Greece

**Keywords:** intraoperative flow cytometry (iFC), glioblastoma, brain metastasis, DNA index, DNA ploidy, cell-cycle analysis, intraoperative diagnosis

## Abstract

Background: Glioblastoma (GBM) and metastatic brain tumours are the two malignant intracranial lesions most frequently encountered in adult neurosurgical practice, and their intraoperative separation may be difficult when imaging is atypical, when no extracranial primary tumour is known, or when frozen-section examination is deferred or inconclusive. Intraoperative flow cytometry (iFC) is an emerging adjunct that provides a rapid, quantitative assessment of tumour DNA content and cell cycle distribution, with a preliminary result available within approximately 5–6 min of tissue receipt. Aim: The aim was to determine, in a preliminary prospective cohort, whether iFC-derived DNA ploidy and cell cycle parameters differ between GBM and brain metastases, and to quantify their discriminatory performance. Methods: iFC was applied to 52 patients operated on for intracranial tumours, including 40 glioblastomas and 12 brain metastases. Tumour samples were analysed for DNA index, G0/G1-phase fraction, and Tumour Index (S + G2/M phases) using the “Ioannina-protocol”. Groups were compared with the Mann–Whitney U test. Discrimination was assessed by receiver operating characteristic (ROC) analysis. Results: Metastases showed a higher DNA index (median 1.05, interquartile range [IQR] 1.00–1.32 versus 1.00, IQR 1.00–1.00; *p* = 0.004) and a higher Tumour Index (24.5%, IQR 19.0–36.0 versus 12.0%, IQR 8.0–19.0; *p* = 0.034), whereas GBM showed a higher G0/G1-phase fraction (88.0%, IQR 81.0–92.0 versus 75.5%, IQR 64.0–81.0; *p* = 0.034). Aneuploidy (DNA index outside 0.9–1.1) was present in 6/12 (50.0%) metastases versus 5/40 (12.5%) GBM (*p* = 0.011). ROC analysis of the Tumour Index yielded an area under the curve of 0.70 (95% CI 0.51–0.88); at the Youden-derived cut-off of 22 the sensitivity was 75.0% (95% CI 42.8–94.5), specificity 75.0% (95% CI 58.8–87.3), positive predictive value 47.4%, negative predictive value 90.9% and overall accuracy 75.0% (95% CI 61.1–86.0). Conclusions: In this small, single-centre preliminary study of 40 glioblastomas and only 12 metastases, iFC detected reproducible differences in DNA ploidy and cell cycle distribution at the group level. Overall discrimination was moderate, so that these parameters cannot at present support an individual-patient intraoperative diagnosis. DNA-based iFC should be regarded as a potential complement to standard intraoperative neuropathological assessment, and requires validation in larger cohorts.

## Simple Summary

This prospective study evaluated the ability of intraoperative flow cytometry (iFC) to differentiate glioblastomas (GBMs) from metastatic brain tumours using DNA ploidy and cell cycle analysis. Fifty-two patients (40 GBMs and 12 metastases) were included. Metastatic tumours demonstrated significantly higher DNA index, Tumour Index, and frequency of DNA aneuploidy, whereas GBMs showed a higher G0/G1-phase fraction. ROC analysis revealed moderate diagnostic performance of the Tumour Index (AUC = 0.70), with 75% sensitivity and specificity at the optimal cut-off. Although iFC identified reproducible group-level differences between the two tumour types, its diagnostic accuracy was insufficient for reliable individual-patient diagnosis. These findings suggest that DNA-based iFC should be considered a rapid complementary intraoperative tool rather than a replacement for conventional neuropathological assessment. Larger prospective validation studies are warranted.

## 1. Introduction

Brain tumours consist of a broad, heterogeneous group of neoplasms that are a significant source of morbidity and mortality worldwide. Approximately two-thirds of brain tumours are secondary, resulting from metastasis of solid tumours, while primary brain tumours account for the remaining one-third of cases [1,2]. Population-based estimates indicate that intracranial metastases develop in approximately 10–30% of adults with systemic malignancy, and their reported incidence has increased over the past two decades as a consequence of improved control of extracranial disease, longer overall survival and the more liberal use of high-resolution magnetic resonance imaging (MRI) for surveillance [3]. Lung and breast cancers are the most frequent sources of intracranial secondary deposits, followed by melanoma, renal cell and colorectal carcinoma [4]. In approximately one in ten patients the intracranial lesion is the first clinical manifestation of an occult extracranial primary tumour [3,4].

Among primary intracranial tumours, meningioma is the most frequently reported histopathology overall, whereas glioblastoma is the most common *malignant* primary brain tumour of adults and ranks third among all histopathologically confirmed primary central nervous system (CNS) neoplasms, after meningioma and tumours of the pituitary region [5]. Glioblastoma has an age-adjusted incidence of approximately three cases per 100,000 person-years, a median age at diagnosis in the seventh decade of life and a modest male predominance [5]. The overwhelming majority of cases are sporadic, no modifiable environmental exposure has been convincingly established, and effective primary prevention is therefore not currently available [6]. Malignant transformation of a previously lower-grade diffuse astrocytoma is well described, but under the 2021 World Health Organization (WHO) classification such tumours are designated astrocytoma, *IDH*-mutant, CNS WHO grade 4, and are regarded as biologically distinct from glioblastoma, *IDH*-wildtype, which is by definition a primary, de novo neoplasm [7].

Surgical resection remains the basis of treatment for patients with primary brain tumours [8]. For newly diagnosed glioblastoma, maximal safe resection is followed by radiotherapy with concomitant and adjuvant temozolomide, with tumour-treating fields and enrolment in clinical trials representing additional options [8,9]. In contrast, the management of brain metastases depends on several parameters, including tumour staging and the patient’s neurological status [10], the number, size and location of the intracranial lesions, the histology and molecular profile of the primary tumour and the presence of extracranial disease; the available modalities comprise surgical resection, stereotactic radiosurgery, whole-brain radiotherapy and, increasingly, CNS-penetrant targeted agents and immune checkpoint inhibitors [10,11]. Regardless of tumour localization, accurate tumour type and grade identification is essential to guide the extent of resection and the therapeutic decisions.

Histopathological evaluation remains the base for tumour diagnosis. Conventional haematoxylin–eosin histology is generally reported within a few working days, whereas the complete integrated report required by the 2021 WHO classification, incorporating *IDH*1/2 status, *ATRX* and p53 immunolabelling, *TERT* promoter mutation analysis, *EGFR* amplification, combined chromosome 7 gain and chromosome 10 loss, and *MGMT* promoter methylation, may take a further two to three weeks, and considerably longer when material must be referred to an external molecular laboratory [7,12]. Intraoperative frozen-section biopsy is a method that offers a relatively fast and accurate method that provides information about tumour type and margins within usually 20 min. Nevertheless, this method has several limitations, including dependency on pathologist expertise and potential diagnostic inaccuracy, especially in infiltrative or heterogeneous tumours [13,14,15,16,17,18,19,20].

Intraoperative flow cytometry (iFC) is an emerging technique that enables rapid assessment, typically less than a minute after sample receipt, of tumour characteristics based on cell cycle distribution and DNA-content analysis. Developed initially as a research tool, iFC has been experimentally applied in various solid organ malignancies [18]. Within the central nervous system (CNS), its application has focused primarily on intracranial tumours, particularly gliomas and meningiomas, where it has shown potential in determining tumour type and grade intraoperatively [19]. Assessment of tumour margins and the presence of neoplastic tissue during stereotactic brain tumour biopsy are additional important applications. We have previously shown that iFC could accurately diagnose and classify central nervous system lymphoma based on immunophenotypic analysis [20].

In technical terms, iFC involves the mechanical dissociation of a small tumour fragment into a single-cell suspension and acquisition on a cytometer, yielding a DNA histogram from which ploidy status and the distribution of nuclei across the G0/G1, S and G2/M phases of the cell cycle can be derived. The principal advantages of the technique are the speed of the assay, its quantitative and largely operator-independent output, the very small amount of tissue consumed, and the fact that the material submitted for permanent histopathology is not compromised. Its limitations are equally explicit: DNA-based iFC yields no morphological, architectural or immunophenotypic information, cannot by itself assign a WHO histological type, is influenced by the proportion of non-neoplastic glial, vascular and inflammatory elements present in the fragment, may be degraded by extensively necrotic or cauterised tissue, and requires a cytometer and appropriately trained personnel to be available within or immediately adjacent to the operating theatre [10,18,19,21,22]. Intraoperative flow cytometry is one of several technologies developed to compress the interval between tissue acquisition and actionable diagnostic information. Alternatives under active investigation include liquid biopsy, stimulated Raman histology and other label-free optical methods, intraoperative mass spectrometry of tumour metabolites, rapid nanopore-based sequencing and intraoperative methylation classification, fluorescence-guided resection using 5-aminolaevulinic acid, and handheld confocal microscopy [12,23,24].

The present study was designed as a prospective analysis aiming to evaluate the utility of flow-cytometric parameters in differentiating glioblastomas (GBMs) from metastatic brain tumours. This discrimination is of substantial intraoperative importance, as it may influence, although it does not by itself determine, the surgical strategy. Brain metastases are frequently amenable to circumferential dissection along a gliotic plane and to en-bloc removal, an approach preferred for selected superficial, non-eloquent lesions in order to limit the risk of leptomeningeal dissemination. However, deep-seated, eloquent or multiple deposits are more often addressed by piecemeal resection, by biopsy alone, or by stereotactic radiosurgery without resection [10,25]. Conversely, surgery for glioblastoma is not confined to cytoreduction: supramarginal resection beyond the contrast-enhancing margin is pursued in selected non-eloquent tumours, whereas stereotactic biopsy alone remains the appropriate procedure for diffusely infiltrating, multifocal or eloquently located disease [8,20,26]. Tumour location and proximity to eloquent cortex and white-matter tracts, the number of lesions, the presence and control status of a known extracranial primary, patient age and performance status, and the preoperative imaging phenotype all contribute to this decision [19,20,27].

Although iFC has been applied to the grading of gliomas and meningiomas and to the intraoperative identification of tumour-infiltrated margins, data on its ability to separate glioblastoma from metastatic brain tumours remain scarce. Because glioblastoma is a diffusely infiltrating primary neoplasm characterised by pronounced intratumoural heterogeneity and by admixture with reactive glial, vascular and inflammatory elements, whereas a metastatic deposit represents a comparatively clonal population of epithelial or melanocytic cells with frequent gross aneuploidy, we hypothesised that the two entities would differ measurably in DNA ploidy and in cell cycle distribution. The specific aims of this preliminary prospective study were therefore threefold: (i) to compare the DNA index, the G0/G1-, S- and G2/M-phase fractions and the Tumour Index between histologically confirmed glioblastoma and brain metastasis; (ii) to quantify the discriminatory performance of the most informative parameter by ROC analysis with internal validation; and (iii) to define, on the basis of the performance actually observed, the realistic position that DNA-based iFC might occupy within the intraoperative diagnostic pathway. The study was explicitly conceived as an exploratory, hypothesis-generating investigation and not as a definitive diagnostic accuracy study intended to establish clinical validity.

## 2. Materials and Methods

### 2.1. Study Design

This prospective cohort study was conducted at the Department of Neurosurgery of the University Hospital of Ioannina during a 4-year period. The study protocol received approval from the Institutional Review Board (IRB) and written informed consent was obtained from all participants (869/6-11-2020). Patients were enrolled consecutively over the study period.

### 2.2. Patient Inclusion and Exclusion Criteria

Inclusion criteria encompassed adult patients (≥18 years) undergoing surgical resection or biopsy for intracranial tumours, with a preoperative diagnosis suggestive of a primary or metastatic brain neoplasm, in whom a specimen of at least 2 mm^3^ could be spared for flow cytometry without compromising the material submitted for histopathology, and in whom a definitive integrated histopathological diagnosis of glioblastoma, IDH-wildtype, or of metastatic carcinoma was subsequently established. Exclusion criteria were: (i) a final histopathological diagnosis other than glioblastoma or metastasis (including *IDH*-mutant astrocytoma, oligodendroglioma, meningioma, and non-neoplastic lesions such as abscess or demyelination); (ii) previous surgery, radiotherapy or chemotherapy directed at the intracranial lesion, in order to avoid the confounding effect of treatment-related changes on the cell cycle distribution; (iii) specimens consisting predominantly of necrotic, haemorrhagic or diathermy-damaged tissue.

### 2.3. Tissue Sampling and Processing

During surgery, tumour samples (~5 mm^3^) were obtained from the tumour core using standard neurosurgical techniques. Half of each sample was sent for routine histopathological examination, while the other half was allocated for intraoperative flow cytometry analysis. Sampling was performed by the operating neurosurgeon at the point of maximal contrast enhancement identified on neuronavigation, immediately after the tumour was entered and before extensive internal debulking, in order to minimise the contribution of thermally damaged tissue. Sharp dissection with a scalpel was used in preference to bipolar diathermy or ultrasonic aspiration. Macroscopically necrotic, cystic and haemorrhagic areas were deliberately avoided. In cases undergoing stereotactic biopsy, one of the sequential core specimens obtained along the trajectory was allocated to iFC and the remainder submitted for histopathology. The specimen designated for iFC was placed dry in a sterile container without fixative or transport medium and hand-carried to the flow cytometry laboratory, situated within the same building, where processing was started within approximately 5 min of excision. The specimen submitted for histopathology was handled according to standard institutional practice and was not affected by the study protocol.

### 2.4. Flow Cytometry Protocol

A standardised intraoperative flow cytometry protocol (Ioannina Protocol), previously developed and optimised for rapid intraoperative analysis, was employed for all included cases [27]. The procedure, lasting approximately less than a minute from tissue receipt to data acquisition, involved processing core tumour specimens (2–5 mm^3^). DNA-content analysis was performed immediately after tumour excision. Tumour samples were thoroughly minced in the Medimachine System (BD Biosciences, Franklin Lakes, NJ, USA) for 1 min in phosphate-buffered saline (free of Ca^2+^ and Mg^2+^, containing 0.5 mg/mL RNase), until a homogeneous cell suspension was obtained. The resulting suspensions were passed through CellTrics mesh filters (Sysmex Flow Cytometry Europe, Norderstedt, Germany) to remove cell aggregates and obtain a single-cell suspension. Cells were counted on an automated haematology analyser and adjusted to a final concentration of 10^6^ cells/mL. The suspension was then processed immediately for staining by the addition of propidium iodide (PI) at a final concentration of 125 μg/mL, and flow-cytometric acquisition was started 3 min later. PI binds stoichiometrically to double-stranded DNA and emits fluorescence upon excitation at 488 nm, with peak emission detected at 617 nm.

### 2.5. Cell Cycle Fractions and Ploidy Status

Flow-cytometric acquisition was performed using a FACSCalibur flow cytometer (BD Biosciences), propidium-iodide fluorescence was acquired through the FL2 detector using linear amplification and data were analysed with CellQuest version 3.1 software (BD Biosciences). Normal peripheral blood mononuclear cells obtained from healthy donors by Ficoll density-gradient separation (Ficoll-Paque, GE Healthcare, Little Chalfont, Buckinghamshire, UK) were used both to calibrate the flow cytometer and to define the diploid G0/G1 peak of the DNA histograms. An average of 5000 events (cell nuclei) was counted and evaluated per sample. A gating strategy based on area/width analysis of propidium iodide fluorescence was applied in order to exclude cell doublets from DNA-content quantification, as previously described. The analysis focused on quantifying the distribution of cells across the G0/G1, S, and G2/M phases of the cell cycle. Two indices were subsequently calculated. The DNA index was defined as the ratio of the geometric mean of the G0/G1 peak of the tumour cells to that of the normal reference cells. A tumour with a DNA index above 1.1 was classified as aneuploid/hyperploid and one with a DNA index below 0.9 as aneuploid/hypoploid, whereas a DNA index of 1 was taken as diploid. The Tumour Index, indicative of tumour cell proliferation, was calculated as the sum of the percentages of cells in the S and G2/M phases.

### 2.6. Histopathological Evaluation

Standard histopathological processing was performed. Tumours were classified according to the World Health Organization (WHO) 2021 classification of central nervous system tumours, determining histological type and grade. The final integrated histopathological diagnosis served as the reference standard throughout and was rendered by neuropathologists who were unaware of the flow-cytometric results.

### 2.7. Statistical Analysis

Continuous variables were expressed as mean ± standard deviation (SD) and, because the distributions were skewed and the group comparisons were performed non-parametrically, as median with interquartile range (IQR); the mean ± SD is retained only to permit comparison with previously published flow-cytometric series, and all inference is based on rank-based methods. Categorical variables were presented as frequencies and percentages. Comparisons between iFC-derived tumour indices were made using the Mann–Whitney U test, for which the U statistic, the two-sided asymptotic *p*-value with correction for ties, and the rank-biserial correlation as a measure of effect size are reported. Categorical variables, including DNA ploidy status, were compared using Fisher’s exact test. Receiver operating characteristic (ROC) curve analysis was employed to evaluate the diagnostic performance of iFC in distinguishing between glioblastomas and metastases. Metastasis was treated as the positive class and the integrated histopathological diagnosis as the reference standard. The area under the ROC curve (AUC) was computed non-parametrically, and its 95% confidence interval (CI) was obtained both by the DeLong method and by percentile bootstrapping with 2000 resamples, the two approaches giving concordant results. The optimal cut-off value for each variable was established using the Youden index (J = sensitivity + specificity − 1), which identifies the threshold that maximises the combined sensitivity and specificity of the test. Sensitivity, specificity, positive and negative predictive values, positive and negative likelihood ratios and overall accuracy at this threshold are reported with exact (Clopper–Pearson) binomial 95% CIs. Because the threshold was derived from, and evaluated in, the same dataset, the resulting estimates are optimistically biased; two internal-validation procedures were therefore applied. First, an optimism-corrected AUC was obtained by the bootstrap method of Harrell with 2000 resamples. Second, the entire procedure, including re-derivation of the Youden threshold, was repeated within a leave-one-out cross-validation loop and within an out-of-bag bootstrap, and the resulting accuracies are reported alongside the apparent values. No adjustment for multiple comparisons was applied, given the exploratory nature of the analysis; the *p*-values should accordingly be interpreted as descriptive rather than confirmatory. A *p*-value < 0.05 was considered statistically significant.

## 3. Results

### 3.1. Patient Characteristics

A total of fifty-two patients (35 men and 17 women, mean age 61.2 years) were included in the analysis, comprising forty cases of glioblastoma (GBM) and twelve cases of metastatic brain tumours. Among the twelve cases of metastatic brain tumours, the primary sites included lung adenocarcinoma (n = 5), lung squamous cell carcinoma (n = 2), breast carcinoma (n = 4), and colorectal carcinoma (n = 1). All forty gliomas were glioblastoma, *IDH*-wildtype, CNS WHO grade 4. The clinical, radiological and operative characteristics of the two groups are summarised in Table 1. Flow-cytometric evaluation included measurement of the DNA index and determination of the proportions of nuclei in the G0/G1, S, and G2/M phases, as well as the calculation of the Tumour Index (S + G2/M).

### 3.2. Cell Cycle Analysis

**Figure 1:**
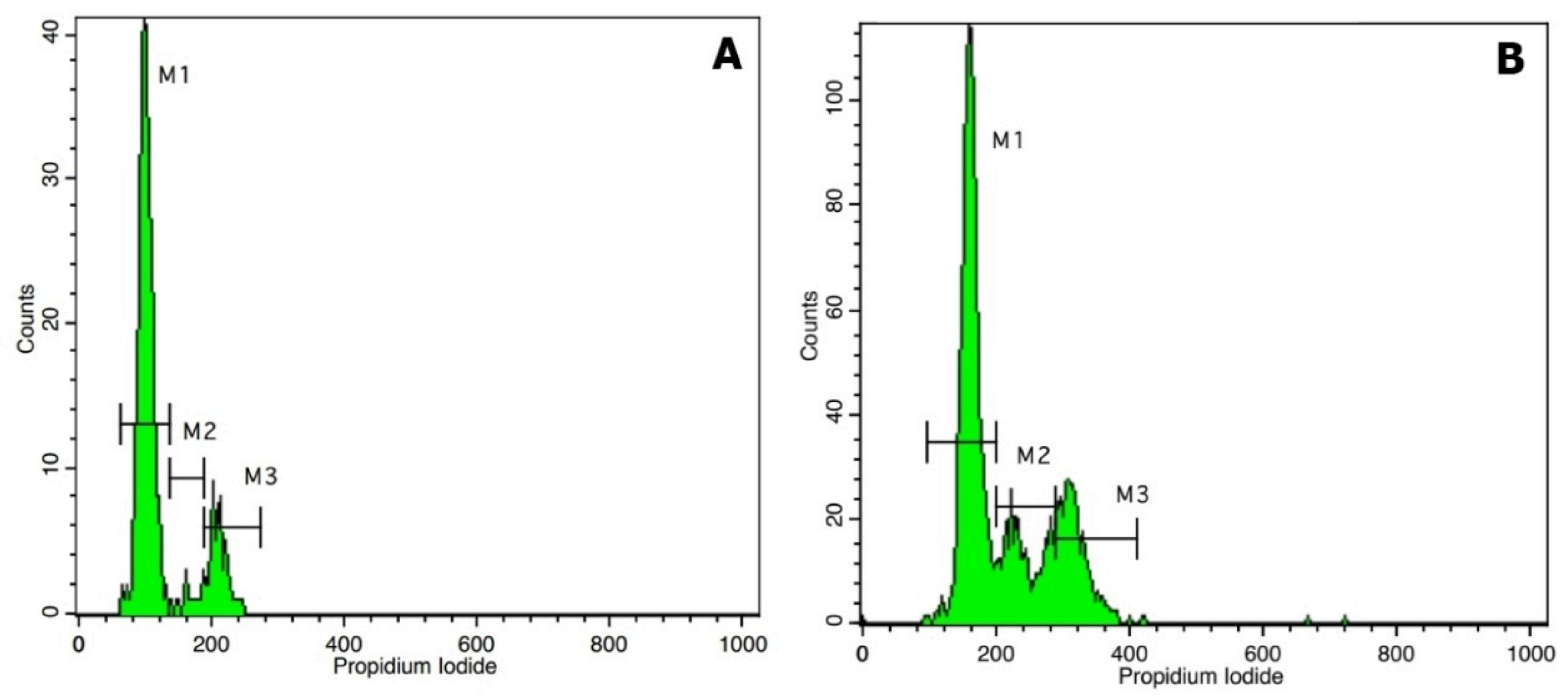
Representative DNA histograms obtained with intraoperative flow cytometry. The horizontal axis shows propidium iodide fluorescence intensity, which is proportional to the DNA content of each nucleus, and the vertical axis shows the number of nuclei recorded at each fluorescence value. Three regions are marked on each histogram: M1 contains resting nuclei with a normal, non-duplicated DNA content (G0/G1 phase); M2 contains nuclei that are actively copying their DNA (S phase); and M3 contains nuclei that have completed DNA duplication and are preparing to divide (G2/M phase). The Tumour Index is the sum of M2 and M3 and therefore represents the proportion of nuclei that are actively cycling. (**A**) DNA analysis with intraoperative flow cytometry in a case of a glioblastoma. Markers M1, M2, and M3 correspond to cells in phase G0/G1, S, and G2/M, respectively. In this case M1 was 82.1%, M2 was 2.8% and M3 was 15.1%. (**B**) A case of metastatic tumour. M1 was 61%, M2 = 19% and M3 = 21%. The metastatic example therefore has a smaller resting population and a Tumour Index of 40%, against 17.9% in the glioblastoma example: the metastasis contains proportionally more dividing cells.

**Figure 2:**
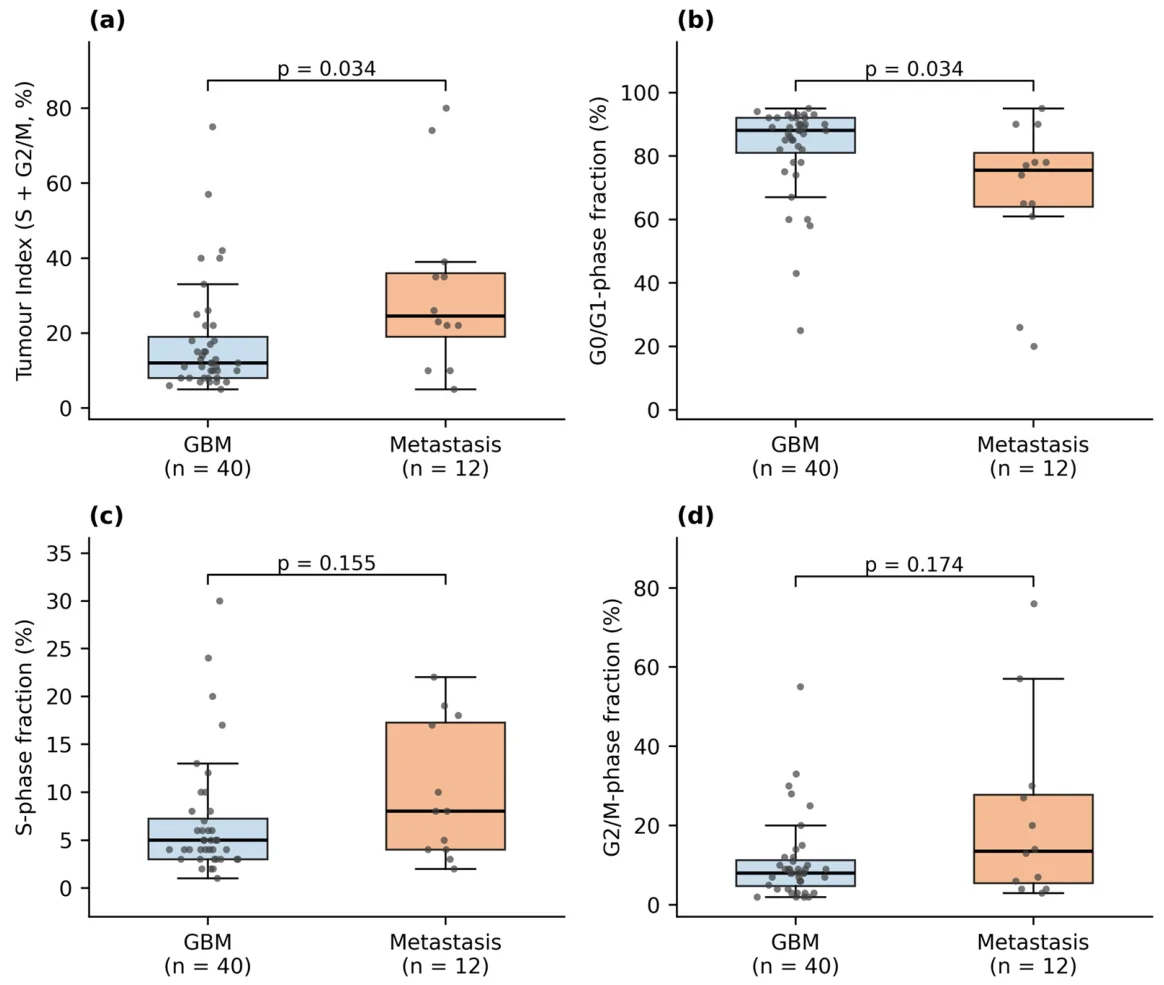
Boxplots demonstrating differences in Tumour Index (**a**), G0/G1-phase fraction (**b**), S-phase fraction (**c**), and G2/M-phase fraction (**d**) between glioblastomas (GBM) and metastatic brain tumours. Glioblastoma n = 40, metastasis n = 12. Each box spans the interquartile range, the horizontal line within the box is the median, and the whiskers extend to the most extreme observation within 1.5 interquartile ranges of the box; every individual patient is additionally plotted as a single point so that the degree of overlap between the two groups can be judged directly. *p*-values are two-sided and derive from the Mann–Whitney U test; the significance threshold was *p* < 0.05. Although the metastatic tumours sit higher than the glioblastomas for the Tumour Index and lower for the G0/G1 fraction, the two distributions overlap substantially, and many individual metastases fall entirely within the glioblastoma range. This overlap is the visual counterpart of the moderate discriminative performance reported in Figure 3.

The G0/G1-phase fraction was greater in GBM (median 88.0%, IQR 81.0–92.0; mean ± SD 82.3 ± 14.8%) than in metastases (median 75.5%, IQR 64.0–81.0; mean ± SD 68.3 ± 23.6%; U = 338.0, *p* = 0.034; rank-biserial r = −0.41). The Tumour Index was higher in metastatic tumours (median 24.5%, IQR 19.0–36.0; mean ± SD 31.8 ± 23.6%) compared with GBM (median 12.0%, IQR 8.0–19.0; mean ± SD 17.7 ± 14.8%; U = 142.0, *p* = 0.034; rank-biserial r = 0.41) [Figure 1]. The DNA index was higher in metastatic tumours (median 1.05, IQR 1.00–1.32; mean ± SD = 1.225 ± 0.322) compared with GBM (median 1.00, IQR 1.00–1.00; mean ± SD 1.065 ± 0.234; U = 144.5, *p* = 0.004; rank-biserial r = 0.40). No statistically significant differences were observed for the S phase (GBM: median 5.0%, IQR 3.0–7.3; mean ± SD 6.8 ± 6.2%; metastases: median 8.0%, IQR 4.0–17.3; mean ± SD 10.0 ± 7.1%; *p* = 0.155) or the G2/M phase (GBM: median 8.0%, IQR 4.8–11.3; mean ± SD 10.9 ± 10.4%; metastases: median 13.5%, IQR 5.5–27.8; mean ± SD 21.8 ± 23.1%; *p* = 0.174). All comparisons are summarised in Table 2. It should be emphasised that the Tumour Index and the G0/G1 fraction are arithmetically complementary, so that these two comparisons are not statistically independent and convey the same underlying information. Boxplots displaying the distribution of the percentages of cell populations in the G0/G1 phase, S phase, G2/M phase and Tumour Index between glioblastomas and metastatic brain tumours are presented in Figure 2.

### 3.3. DNA Ploidy Status

When DNA content was analysed as a categorical variable, a DNA-aneuploid pattern (DNA index < 0.9 or >1.1) was identified in six of 12 metastatic tumours (50.0%) compared with five of 40 glioblastomas (12.5%; Fisher’s exact test, *p* = 0.011). Expressed differently, a DNA-diploid histogram was obtained in 87.5% of glioblastomas, so that the finding of overt aneuploidy in an intraoperative specimen shifted the diagnosis towards a metastatic lesion, whereas a diploid histogram was uninformative because it occurred in half of the metastases as well. Given the very small number of metastatic cases, this observation should be treated as preliminary.

### 3.4. Diagnostic Performance of the Tumour Index

**Figure 3:**
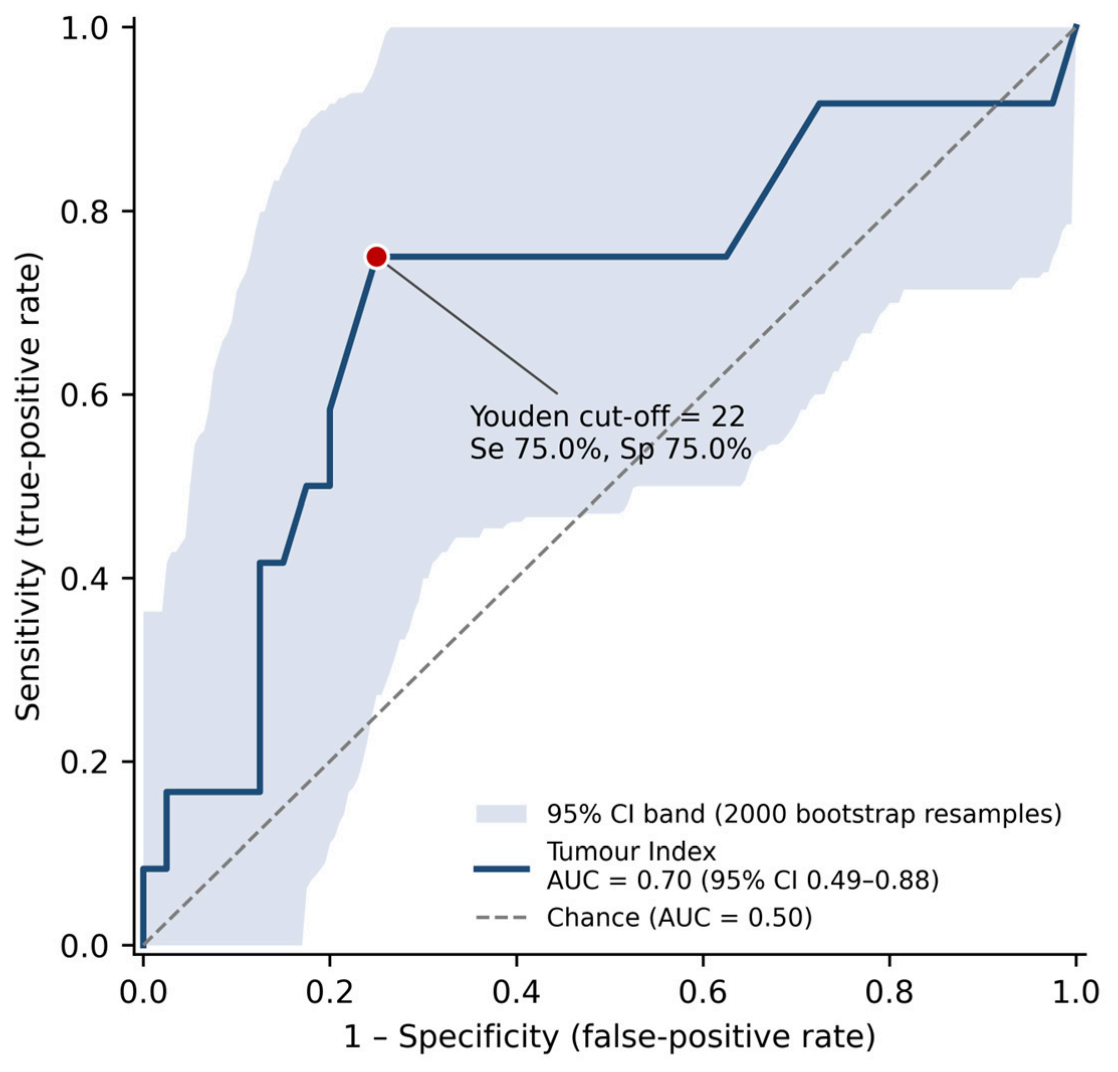
ROC analysis of the Tumour Index for the discrimination of metastatic brain tumours from glioblastomas (n = 52; 12 metastases, 40 glioblastomas). The curve shows, for every possible Tumour Index threshold, the proportion of metastases correctly identified (sensitivity, vertical axis) against the proportion of glioblastomas incorrectly labelled as metastases (1 − specificity, horizontal axis). A test with no discriminating ability would follow the diagonal dashed line. The shaded band is the 95% confidence region obtained from 2000 bootstrap resamples and illustrates the uncertainty that arises from the small number of metastatic cases; because this band includes the diagonal over much of its course, the possibility that the true performance is close to chance cannot be excluded. The red point marks the threshold selected by the Youden index (Tumour Index = 22%), at which sensitivity and specificity were both 75.0%. AUC = 0.70 (95% CI 0.51–0.88).

Receiver operating characteristic (ROC) curve analysis was performed for the Tumour Index (Figure 3). The Tumour Index yielded an AUC of 0.70 (95% CI 0.51–0.88) for discriminating metastases from GBM. Using a cut-off value of 22, based on Youden’s index, the sensitivity was 75.0% (9/12, 95% CI 42.8–94.5), specificity 75.0% (30/40, 95% CI 58.8–87.3), and overall accuracy 75.0% (39/52, 95% CI 61.1–86.0). At the prevalence of metastasis observed in this cohort (23.1%), the positive predictive value was 47.4% (95% CI 24.4–71.1) and the negative predictive value 90.9% (95% CI 75.7–98.1), with a positive likelihood ratio of 3.0 and a negative likelihood ratio of 0.33. In other words, of the 19 specimens classified as metastatic by the Tumour Index, 10 were glioblastomas. Because the cut-off was both derived and evaluated in the same 52 patients, these figures are optimistically biased. Bootstrap optimism correction changed the AUC only marginally (optimism-corrected AUC 0.70), but re-deriving the threshold within a leave-one-out cross-validation loop gave an accuracy of 75.0%, and an out-of-bag bootstrap procedure gave a mean accuracy of 72.8% (95% CI 55.6–88.2), the lower bound of which approaches the accuracy obtainable by classifying every specimen as glioblastoma (76.9%). Diagnostic performance measures are summarised in Table 3. Exploratory ROC analysis of the DNA index yielded a comparable AUC of 0.70 (95% CI 0.55–0.85), with a sensitivity of 50.0% and a specificity of 90.0% at a cut-off of 1.1; no combination of parameters was modelled, since the number of metastatic events (n = 12) is far below that required for multivariable estimation.

## 4. Discussion

### 4.1. Main Findings

The present study evaluated the potential role of iFC in differentiating GBM from metastatic brain tumours based on cell cycle distribution and DNA content. Our results demonstrated significant differences in the DNA index, G0/G1-phase fraction, and Tumour Index between the two tumour types. Specifically, GBMs exhibited higher proportions of cells in the G0/G1 phase, while metastatic tumours showed increased Tumour Index and higher DNA index values, indicative of greater aneuploidy and proliferative activity. ROC analysis revealed that a Tumour Index cut-off value of 22 achieved an overall accuracy of 75% for distinguishing between GBM and metastasis. These differences were, however, demonstrated at the level of the group and not of the individual patient. The area under the curve of 0.70 denotes moderate discrimination only, its confidence interval extends to 0.51, and the positive predictive value at the observed prevalence was below 50%, meaning that most specimens flagged as metastatic by the Tumour Index were in fact glioblastomas. The overall accuracy of 75% must also be read against the accuracy of 76.9% that would be obtained by classifying every specimen as glioblastoma in a cohort with this case mix. The negative predictive value of 90.9% is the more robust figure, and the practical implication is that a low Tumour Index argues moderately against a metastatic lesion, whereas a high Tumour Index is too non-specific to act upon in isolation.

### 4.2. Biological Basis of the Observed Differences

At a cellular level, the differences observed in DNA index, G0/G1 fraction, and Tumour Index between glioblastomas and metastatic tumours may reflect their distinct biological behaviour. Glioblastomas are highly heterogeneous, containing both rapidly dividing and quiescent cell populations, as well as reactive glial and inflammatory elements, which could explain the higher G0/G1 fraction and lower overall proliferative activity detected by iFC [21,28]. In contrast, metastatic tumours often consist of more homogeneous, clonally derived cells with greater chromosomal instability and a higher proportion of actively cycling cells, resulting in increased aneuploidy and Tumour Index values [29,30]. It is important to state explicitly that none of these mechanisms was tested in the present dataset. We did not quantify tumour cell purity, did not perform immunophenotyping to separate neoplastic from reactive populations, and did not obtain copy-number or karyotypic data; the biological account offered here is therefore an interpretation consistent with the observed histograms rather than a demonstrated explanation.

Overall, the loss of cell cycle control that underlies an elevated Tumour Index is mechanistically well characterised in both entities. In glioblastoma, homozygous deletion of CDKN2A/B removes p16INK4a-mediated inhibition of the CDK4/6–cyclin D complex, RB1 alteration releases the G1/S restriction point, and amplification or mutation of EGFR with downstream PI3K–AKT–mTOR activation sustains proliferative signalling; TP53 pathway disruption in turn permits the propagation of cells carrying unrepaired DNA damage [31]. The consequence at the level of a DNA histogram is an increased S and G2/M fraction. That glioblastomas in the present series nevertheless showed a lower Tumour Index than metastases is best explained not by a lower intrinsic proliferative rate of the neoplastic clone but by the composition of the analysed specimen: a glioblastoma fragment contains a substantial and variable admixture of non-neoplastic diploid cells ll of which reside in G0/G1 and dilute the cycling fraction. A metastatic deposit, growing as a comparatively circumscribed and clonal population, contains proportionally fewer such cells [32]. An analogous argument applies to DNA content. Aneuploidy, defined cytometrically as a DNA index outside the diploid range, arises from chromosomal instability, from defects in the spindle-assembly checkpoint and centrosome number, and frequently from whole-genome doubling, and is tolerated in cancer cells through adaptive changes in the p53 pathway [33]. Glioblastoma, IDH-wildtype, is typically characterised by recurrent, focal copy-number alterations which alter the genome substantially in biological terms but change the total nuclear DNA content comparatively little, so that the histogram often remains near-diploid. Many carcinomas, by contrast, exhibit whole-genome doubling and large-scale numerical aberrations that shift the modal DNA content well beyond the diploid range [31,34]. This is consistent with our observation that 87.5% of glioblastomas were DNA-diploid whereas half of the metastases were aneuploid, and it also explains why a diploid histogram carries little diagnostic weight: it remains common in metastasis. We emphasise, however, that the proportion of glioblastomas classified as aneuploid varies markedly between published iFC series, so that the ploidy distribution reported here should not be assumed to generalise to other laboratories or protocols [35].

### 4.3. Intraoperative Flow Cytometry Compared with Conventional Intraoperative Diagnosis

In recent years, flow cytometry has been preliminary applied to the evaluation of various solid tumours, including primary brain neoplasms, head and neck cancers, and tumours of the breast, colorectal, and urogenital systems [36,37]. Its potential utility lies in complementing intraoperative frozen section by providing rapid, quantitative information that enhances diagnostic accuracy and assists in assessing tumour margins, which may ultimately determine the extent of resection. This is particularly important in high-grade gliomas, where the surgical goal is to achieve maximal safe cytoreduction while preserving eloquent brain areas and maintaining the patient’s quality of life [8]. In contrast, metastatic brain tumours typically exhibit well-defined margins, allowing for “*en-bloc*” resection with the aim of complete removal.

Frozen-section examination and smear or squash cytology remain the reference intraoperative techniques and are the only ones that provide architectural and cytological information; they can recognise glandular formation, keratinisation, melanin pigment, palisading necrosis and microvascular proliferation, and can therefore assign an entity rather than a probability. Their disadvantages are a turnaround of 20–30 min or more, dependence on the availability and experience of a neuropathologist, and the consumption of tissue. Intraoperative immunohistochemistry adds specificity but is rarely feasible within an operative timeframe. Against this background, iFC offers a result in under a minute, an objective numerical output that does not depend on the interpretation of morphology, a tissue requirement of only a few cubic millimetres, and the ability to be repeated at several sites within the same operation, for example along a resection margin, at negligible marginal cost. Its corresponding weakness is that it measures only two quantities, DNA content and cell cycle distribution, neither of which is disease-specific; it cannot identify the tissue of origin of a metastasis, cannot recognise entities such as lymphoma or abscess without the addition of immunophenotyping, and, as our data show, produces distributions that overlap considerably between glioblastoma and metastasis. The reasonable conclusion is that iFC is complementary to, and cannot substitute for, neuropathological assessment; its most plausible role is as a rapid, quantitative adjunct available in the interval before the frozen-section result, and as a means of interrogating multiple sites when the frozen section is deferred or inconclusive [18,19,22,38].

However, the application of frozen-section analysis and gross macroscopic evaluation of tumour features may still result in a considerable diagnostic discordance rate, with misclassification or deferral reported in up to 20–25% of brain tumour cases [14,15]. In such instances, determining the optimal surgical strategy can be challenging and may ultimately rely on the surgeon’s intraoperative judgement, experience, and the patient’s or family’s preferences regarding postoperative quality of life [15]. Thus, the introduction of novel efficient intraoperative methods could help to minimise this rate of diagnostic errors. In our preliminary study, the use of cell cycle parameters resulted in observable differences between glioblastomas and metastatic brain tumours, with Tumour Index resulting in approximately 75% specificity and sensitivity. The latter could be further improved with the addition of phenotypic analysis in future studies that will include clusters of differentiation [19,20]. Taken together, these observations suggest that DNA-based iFC performs better when the contrast being drawn is one of proliferative activity within a single tumour lineage—low-grade versus high-grade glioma—than when it is asked to separate two lineages that may share a similar proliferative phenotype, as glioblastoma and metastatic carcinoma frequently do. This is a coherent explanation of why the present sensitivity and specificity are lower than those reported for glioma grading, and it argues that immunophenotypic markers, rather than additional cell cycle parameters, are the logical next step [38]. Our findings are concordant with the only previous observation on this question, a communication from our own group reporting that metastatic brain tumours show a lower G0/G1 fraction and higher S-phase and mitotic fractions than primary brain tumours [39]. A recent systematic review of iFC studies confirms that, beyond that single report, evidence on brain metastases is essentially absent [39]. However, Koriyama et al., using a comparable rapid intraoperative protocol in glioblastomas, detected DNA aneuploidy in 54.5% of cases, a proportion more than four times higher than the 12.5% observed here, and described glioblastoma as a predominantly aneuploid tumour [35]. It is most plausibly explained by differences in mechanical dissociation and gating, in the fluorescence threshold at which a second peak is called aneuploid, and in the proportion of non-neoplastic diploid nuclei retained in the analysed suspension, and it is a direct argument for the standardisation of iFC protocols before DNA ploidy is used as a diagnostic criterion [38].

### 4.4. Novelty and Clinical Positioning

The novelty of the present work lies less in the technique itself, which our group and others have described previously, rather than in the specific diagnostic question addressed and in the way the result is reported. A recent systematic review of intraoperative flow cytometry across surgical oncology found that its application to brain metastases remains confined to correspondence reporting small numbers of cases, with no prospective series and no proposed threshold [39]. To our knowledge the present work is therefore the first prospective study to apply rapid intraoperative DNA-content and cell cycle analysis specifically to the glioblastoma-versus-metastasis distinction, to derive and internally validate a numerical threshold for that distinction, and to report the ploidy status of the two entities as a categorical variable.

Accurate identification of tumour type also has consequences beyond the operation itself, because it determines the postoperative imaging strategy and the framework within which subsequent lesions are interpreted. The differentiation of true tumour recurrence from treatment-related change is a recurring problem in this population, and functional imaging modalities such as positron emission tomography and single-photon emission tomography have been proposed as adjuncts to conventional neuroradiology for exactly this purpose, including in the distinction of postoperative change from recurrent neoplasm [40].

Future studies should be prospectively designed to directly compare the diagnostic accuracy of frozen-section biopsy with that of iFC and to develop integrated diagnostic algorithms that combine both methods. The most immediate priority is a head-to-head design in which the frozen-section diagnosis, the iFC result and the final integrated histopathological diagnosis are recorded prospectively for every patient, allowing the incremental value of iFC over frozen section, and the performance of the two in combination, to be quantified rather than assumed. A second priority is the addition of a short immunophenotypic panel to the DNA protocol, since surface and intracellular markers capable of separating glial from epithelial lineages would address the principal weakness identified here. Third, any threshold proposed for clinical use must be derived in one cohort and tested in a separate, preferably multicentre, validation cohort with a metastatic group large enough to permit stratification by primary tumour site. Ultimately, the implementation of iFC in the operating room has the potential to reduce intraoperative uncertainty and to support individualised surgical strategies, provided that its performance characteristics are established with the rigour that any diagnostic test intended to influence an operation requires.

### 4.5. Limitations

The present study has several limitations. First, the sample size was relatively small, especially for the group of metastatic tumours: only 12 metastatic cases were available, which is the dominant source of statistical uncertainty in this work and is directly responsible for the width of the confidence interval around the area under the curve (0.51–0.88). With 12 events, the study was powered to detect only large differences, and all estimates of diagnostic performance should be regarded as unstable. A prospective comparison of diagnostic accuracy with frozen section was not performed; the intraoperative frozen-section diagnosis was not recorded systematically for each patient, and we are therefore unable to state whether iFC would have added anything to, or agreed with, the intraoperative neuropathological assessment. This is the most important omission of the study and the principal reason why the clinical utility of the method remains unproven rather than merely uncertain. In addition, the metastatic cases came from different primary cancers, each of which can have distinct biological and proliferative behaviours. This heterogeneity could have influenced the variability in the flow-cytometric results; lung adenocarcinoma, squamous cell carcinoma, breast carcinoma and colorectal carcinoma differ appreciably in ploidy and proliferative fraction, and with one to five cases per primary site no stratified analysis was possible. The metastatic group should therefore be understood as a convenience aggregate rather than as a biologically coherent category, and the pooled median Tumour Index reported here may not apply to any individual primary tumour type. Another limitation is that intraoperative flow cytometry was based solely on DNA-content and cell cycle analysis, and a phenotypic analysis was not performed. Sampling bias was not formally assessed, since the analysed specimen might not always represent the full heterogeneity of a glioblastoma; a single fragment taken from the tumour core cannot capture the regional variation in proliferative activity that characterises glioblastoma, and sampling from the infiltrative margin or from a different quadrant might well have produced different values; however, there was always histopathological evaluation of the other half of the specimen. Furthermore, the Tumour Index threshold of 22 was derived and evaluated in the same 52 patients using the Youden index, which is known to yield optimistically biased estimates; although bootstrap and leave-one-out internal validation suggested that the optimism was modest in this instance, internal validation cannot substitute for testing in an independent cohort, and no external validation was undertaken. The single-centre design, the use of a single cytometer and a single pair of experienced operators further limit generalisability, and inter-laboratory reproducibility of the Tumour Index has not been established. Finally, the case mix of the cohort, 77% glioblastoma, determines the predictive values reported here, which would differ in a population with a different prevalence of metastatic disease. Finally, combining iFC results with molecular or immunohistochemical markers in future studies could further improve diagnostic precision and clinical utility.

## 5. Conclusions

This preliminary study indicates that intraoperative flow cytometry can detect group-level differences between glioblastomas and metastatic brain tumours in DNA content and cell cycle distribution. Metastatic tumours showed higher DNA index and Tumour Index values and a higher frequency of DNA aneuploidy, whereas glioblastomas showed a greater proportion of nuclei in the G0/G1 phase. Discrimination was nonetheless only moderate: the Tumour Index achieved an area under the curve of 0.70 with a confidence interval extending close to chance, sensitivity and specificity of 75%, and a positive predictive value below 50% at the prevalence observed in this cohort. These parameters are therefore not sufficient to establish an intraoperative diagnosis in an individual patient, and DNA-based intraoperative flow cytometry should be regarded as a rapid quantitative adjunct to, and not a substitute for, intraoperative neuropathological assessment. Its most defensible current use is to provide, within one minute of tissue receipt, a probabilistic indication that is available before the frozen-section report and that may be repeated at multiple sites. Larger prospective cohorts with an adequately sized and stratified metastatic group, a head-to-head comparison with frozen section, the addition of immunophenotypic markers, and external validation of any proposed threshold are all required before this technique can be considered for routine intraoperative use.

## Figures and Tables

**Table 1:** Clinical, radiological and operative characteristics of the study population.

Characteristic	Glioblastoma (n = 40)	Metastasis (n = 12)	Overall (n = 52)
**Age** **, years (mean ± SD)**	**59.6 ± 11.8**	**66.5 ± 9.4**	**61.2 ± 11.5**
**Sex**			
Male	27 (67.5%)	8 (66.7%)	35 (67.3%)
Female	13 (32.5%)	4 (33.3%)	17 (32.7%)
**Tumour location**			
Frontal	16 (40.0%)	5 (41.7%)	21 (40.4%)
Temporal	11 (27.5%)	3 (25.0%)	14 (26.9%)
Parietal	9 (22.5%)	3 (25.0%)	12 (23.1%)
Occipital	4 (10.0%)	1 (8.3%)	5 (9.6%)
**Pathology**	All IDH-wildtype, CNS WHO grade 4	Lung adenocarcinoma (n = 5), lung squamous cell carcinoma (n = 2), breast carcinoma (n = 4), colorectal carcinoma (n = 1)	GBM: 40 cases (76.9%); Metastasis: 12 cases (23.1%)

**Table 2:** Intraoperative flow-cytometric parameters in glioblastoma (n = 40) and metastatic brain tumours (n = 12). Values are median (IQR) with mean ± SD in parentheses. *p*-values are two-sided and derive from the Mann–Whitney U test with correction for ties; the significance threshold was *p* < 0.05.

Parameter	Statistic	GBM (n = 40)	Metastasis (n = 12)	U	*p*
DNA index	Median (IQR)	1.00 (1.00–1.00)	1.05 (1.00–1.32)	14 4.5	0.004
	Mean ± SD	1.065 ± 0.234	1.225 ± 0.322		
G0/G1 phase, %	Median (IQR)	88.0 (81.0–92.0)	75.5 (64.0–81.0)	338.0	0.034
	Mean ± SD	82.3 ± 14.8	68.3 ± 23.6		
S phase, %	Median (IQR)	5.0 (3.0–7.3)	8.0 (4.0–17.3)	174.5	0.155
	Mean ± SD	6.8 ± 6.2	10.0 ± 7.1		
G2/M phase, %	Median (IQR)	8.0 (4.8–11.3)	13.5 (5.5–27.8)	177.0	0.174
	Mean ± SD	10.9 ± 10.4	21.8 ± 23.1		
Tumour Index (S + G2/M), %	Median (IQR)	12.0 (8.0–19.0)	24.5 (19.0–36.0)	142.0	0.034
	Mean ± SD	17.7 ± 14.8	31.8 ± 23.6		

Abbreviations: GBM, glioblastoma; IQR, interquartile range; SD, standard deviation.

**Table 3:** Diagnostic performance of the Tumour Index for the identification of metastatic brain tumours (positive class), at the Youden-derived cut-off of 22, in 52 patients (12 metastases, 40 glioblastomas). Proportions are given with exact (Clopper–Pearson) binomial 95% confidence intervals.

Measure	Value (95% CI)
Area under the ROC curve	0.70 (0.51–0.88)
Optimism-corrected AUC (bootstrap, 2000 resamples)	0.70
Cut-off (Youden index)	Tumour Index ≥ 22%
Sensitivity	75.0% (42.8–94.5); 9/12
Specificity	75.0% (58.8–87.3); 30/40
Positive predictive value (prevalence 23.1%)	47.4% (24.4–71.1); 9/19
Negative predictive value (prevalence 23.1%)	90.9% (75.7–98.1); 30/33
Overall accuracy	75.0% (61.1–86.0); 39/52
Accuracy, leave-one-out cross-validation	75.0%

AUC, area under the curve; CI, confidence interval; GBM, glioblastoma; ROC, receiver operating characteristic.

## Data Availability

The data presented are available upon request from the corresponding author.

## Abbreviations

AUC, area under the receiver operating characteristic curve; CI, confidence interval; CNS, central nervous system; CT, computed tomography; GBM, glioblastoma; iFC, intraoperative flow cytometry; IDH, isocitrate dehydrogenase; IQR, interquartile range; MGMT, O6-methylguanine-DNA methyltransferase; MRI, magnetic resonance imaging; PI, propidium iodide; ROC, receiver operating characteristic; SD, standard deviation; WHO, World Health Organization.
